# Supplementary material for: Glyoxalase-1-Dependent Methylglyoxal Depletion Sustains PD-L1 Expression in Metastatic Prostate Cancer Cells: A Novel Mechanism in Cancer Immunosurveillance Escape and a Potential Novel Target to Overcome PD-L1 Blockade Resistance
Source: Cancers (Basel). 2021 Jun 13;13(12):2965. doi: 10.3390/cancers13122965 (PMC8232032; doi:10.3390/cancers13122965)
Supplement: Supplementary file 1 [file cancers-13-02965-s001.zip › cancers-1253786-supplementary.pdf]

Supplementary Material: Glyoxalase 1-dependent methylglyoxal depletion sustains PD-L1 expression in metastatic prostate cancer cells: a novel mechanism in cancer immunosurveillance escape and a potential novel target to overcome PD-L1 blockade resistance

Cinzia Antognelli, Martina Mandarano, Enrico Prosperi, Angelo Sidoni, and Vincenzo Nicola Talesa

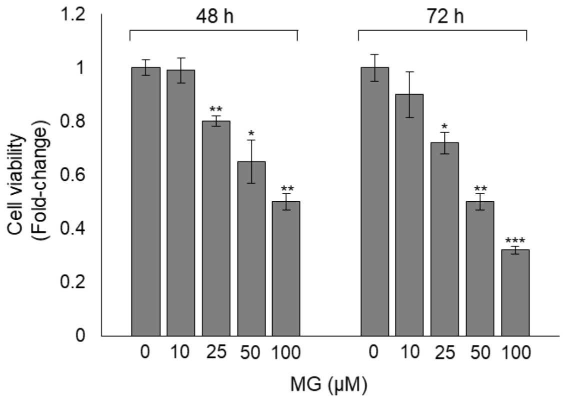

(a)

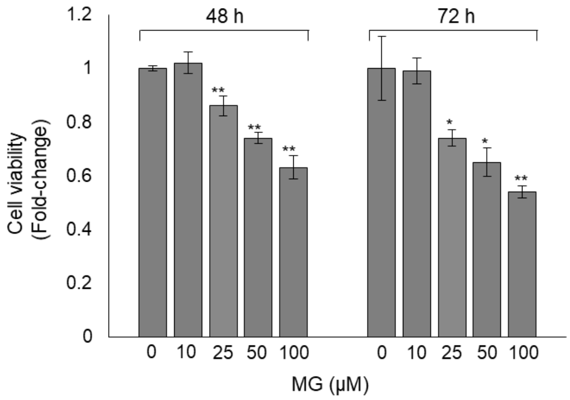

(b)

Figure S1. Methylglyoxal (MG) effect on (a) DU-145 and (b) PC3 viability unexposed or exposed for 48 and 72 hours at the different indicated concentrations. Cell viability was measured by MTT assay. Data report the means of three separate experiments performed in duplicate and error bars represent the standard deviation (SD) of the mean. *p < 0.05, **p < 0.01 and ***p < 0.001 significantly different from untreated control cells.

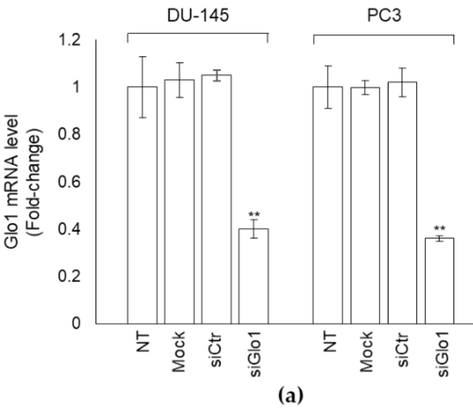

(a)

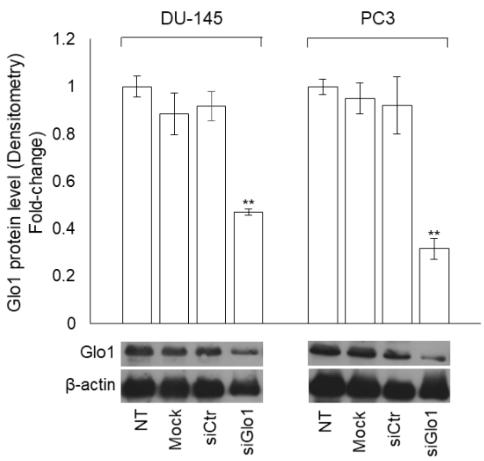

(b)

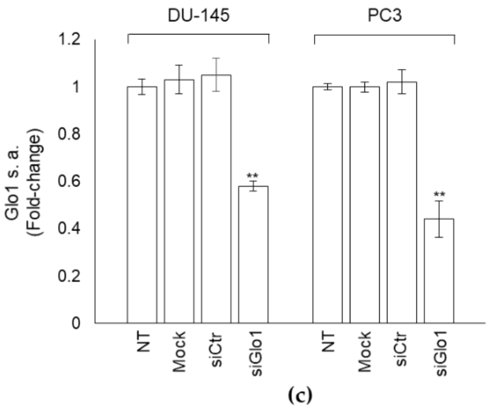

(c)

Figure S2. Glyoxalase 1 (Glo1) silencing by small interfering RNA in DU-145 and PC3 cells. Cells were transfected for 72 hours with vehicle alone (Mock), control non-targeting siRNA (siControl, siCtr) and Glo1-siRNA (siGlo1) or non-transfected (NT). (a) Glo1 mRNA expression was detected by qRT-PCR; (b) Glo1 protein level was evaluated by western

blot according to what described in M&M with 60 μg of proteins; (c) Glo1 enzymatic specific activity was detected by a spectrophotometric method. All the data are means ± SD of three independent experiments done in duplicate. **p < 0.01 significantly different from untreated control cells.

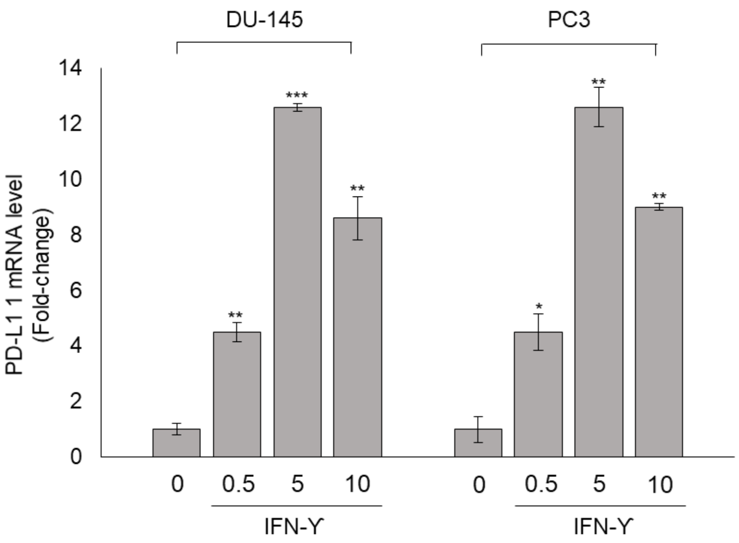

Figure S3. Effect of IFN-γ on PD-L1 transcript level in DU-145 and PC3 metastatic prostate cancer cells. Cells were exposed for 8 hours at the different indicated concentrations of IFN-γ and PD-L1 mRNA levels were measured by qRT-PCR. Data report the means of three separate experiments performed in duplicate and error bars represent the standard deviation (SD) of the mean. *p < 0.05, **p < 0.01 and ***p < 0.001 significantly different from untreated control cells.

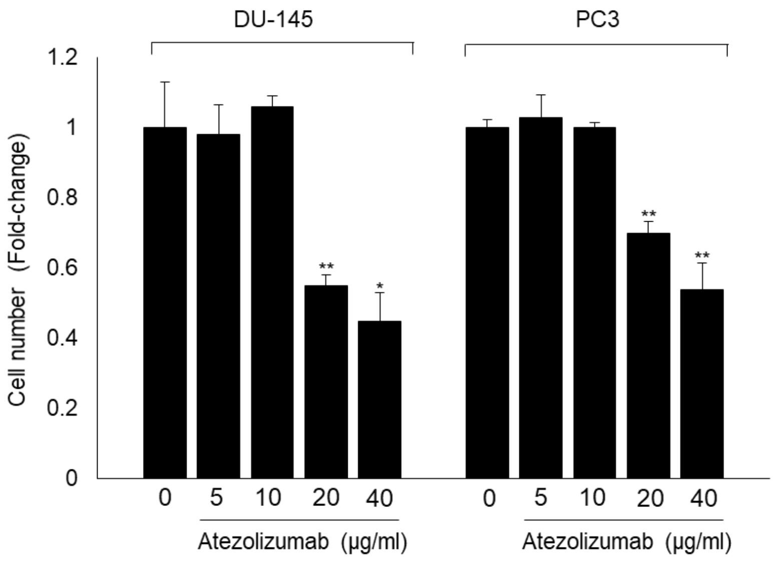

Figure S4. Effect of Atezolizumab on DU-145 and PC3 metastatic prostate cancer cell number. Cells were exposed at the different indicated concentrations of atezolizumab and cell number was measured by cell counting. Data report the means of three separate experiments performed in duplicate and error bars represent the standard deviation (SD) of the mean. *p

< 0.05 and **p < 0.01 significantly different from untreated control cells. Control IgG isotype did not affect DU-145 and PC3 cell number (data not shown)

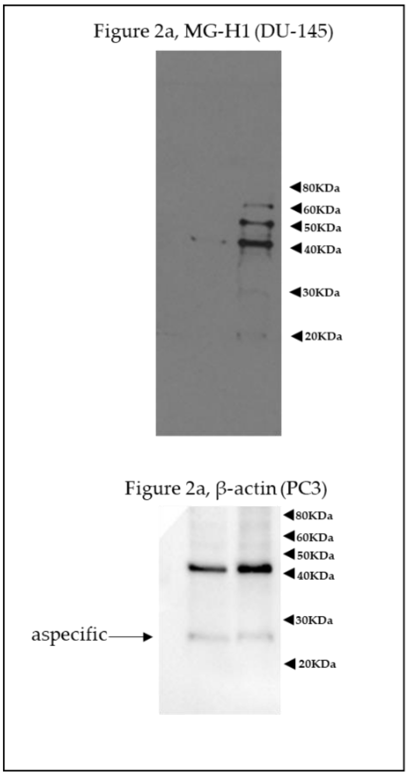

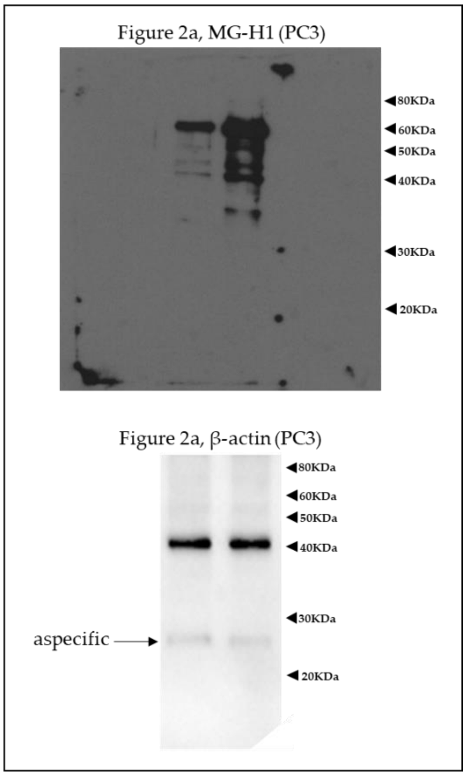

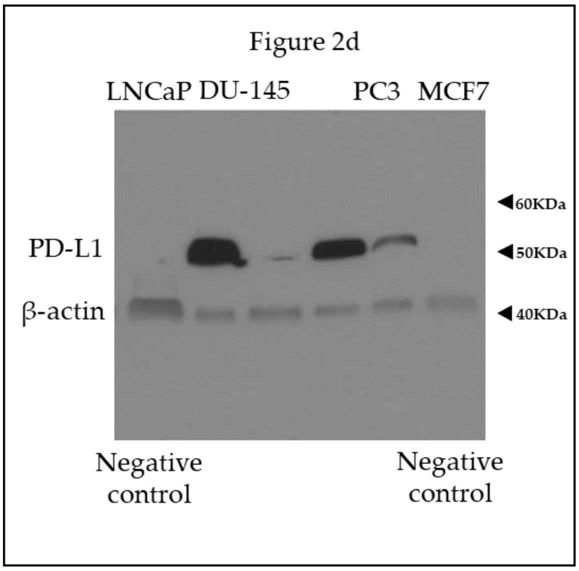

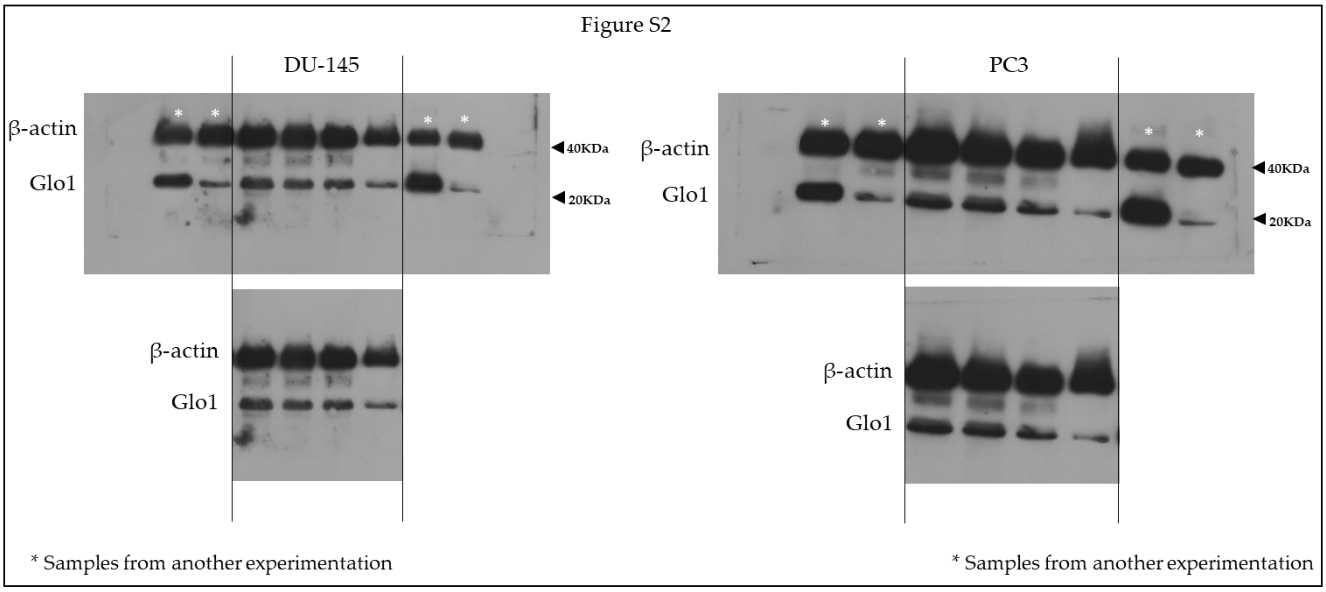

Figure S5. Whole blots reported in Figure 2 and Figure S2.
